# Comparative genomics meets topology: a novel view on genome median and halving problems

**DOI:** 10.1186/s12859-016-1263-7

**Published:** 2016-11-11

**Authors:** Nikita Alexeev, Pavel Avdeyev, Max A. Alekseyev

**Affiliations:** The George Washington University, Washington, DC USA

**Keywords:** Median problem, Halving problem, Breakpoint graphs, Embedded graphs

## Abstract

**Background:**

Genome median and genome halving are combinatorial optimization problems that aim at reconstruction of ancestral genomes by minimizing the number of evolutionary events between them and genomes of the extant species. While these problems have been widely studied in past decades, their solutions are often either not efficient or not biologically adequate. These shortcomings have been recently addressed by restricting the problems solution space.

**Results:**

We show that the restricted variants of genome median and halving problems are, in fact, closely related. We demonstrate that these problems have a neat topological interpretation in terms of embedded graphs and polygon gluings. We illustrate how such interpretation can lead to solutions to these problems in particular cases.

**Conclusions:**

This study provides an unexpected link between comparative genomics and topology, and demonstrates advantages of solving genome median and halving problems within the topological framework.

## Introduction

One of the key computational problems in comparative genomics is the reconstruction of ancestral genomes based on gene^1^ orders in the extant species [1–4]. Since most dramatic changes in genomic architectures are caused by *genome rearrangements* (such as *reversals*, *translocations*, *fusions*, and *fissions*), this problem is often posed as minimization of the total *distance* (i.e., the number of genome rearrangements) between extant and ancestral genomes along the branches of the evolutionary tree. The basic case of three given genomes represents the *genome median problem* (GMP), which asks for reconstruction of a single ancestral genome, called *median genome*.

Since genome rearrangements preserve the gene content, it must be restricted to genes present in all input genomes with the same multiplicity. To account for genes appearing different number of times in different genomes, one need to consider other types of evolutionary events. One of important sources of duplicated genes in genomes are the *whole genome duplication* (WGD) events that simultaneously duplicate each chromosome of a genome. WGD events are known to happen in evolution of yeasts [5], fishes [6], plants [7], and even mammalian species [8], which inspires the problem of reconstruction of *doubled genomes*, i.e., genomes immediately resulted from a WGD in the course of evolution. This problem is often posed for input genomes that have all genes present either in a single copy (*ordinary genomes*) or in two copies (*all-duplicated genomes*). In the simplest form, it is known as the *genome halving problem* (GHP), which asks for an ordinary genome for a given all-duplicated genome such that the distance between them is minimized. In the case of a given all-duplicated genome and an ordinary genome, the problem, called the *guided genome halving problem* (GGHP), asks for an ordinary genome at the minimal total distance from both given genomes.

While the GHP admits a polynomial solution [9–11], its solution space is enormously large, which makes it impractical to obtain biologically adequate doubled genomes. The GGHP improves biological relevance by using an additional ordinary genome. Similarly, solutions for the GMP are not always biologically adequate [12–14]. Furthermore, the GGHP and GMP are known to be NP-complete in many models of genome rearrangements. This obstacles inspire researchers to study restricted variants of the GGHP and GMP.

A recently introduced variant of the GMP, called the *intermediate genome median problem* (IGMP), restricts its solutions to the *intermediate genomes*, i.e., genomes appearing in a shortest rearrangement scenario between two of the three given genomes [13]. Similarly, for the GGHP, there exists a variant (we called it the *restricted guided genome halving problem*, RGGHP) that restricts the constructed doubled genomes to the GHP solution space [15]. It is worth to mention that the proposed heuristic solutions [13, 15] to the IGMP and RGGHP are based on similar ideas. We also remark that the computational complexity of these problems remain an open question.

In this study, we show that the IGMP and RGGHP are, in fact, closely related, and put them into the framework of embedded graphs and polygon gluings [16]. This framework is traditionally studied in mathematical physics and has applications in fields such as random matrices [17] and moduli space of curves [18]. It is also studied in computational geometry with applications in computer graphics and related fields [19, 20]. More recently, it has been also applied in computational biology for analysis of RNA secondary structure [21, 22]. We show that the topological reformulation of the IGMP and RGGHP leads to solving these problems in some particular cases. As a by-product, we also determine the cardinality of the GHP solution space.

## Background

### Genome rearrangements and breakpoint graphs

**Fig. 1:**
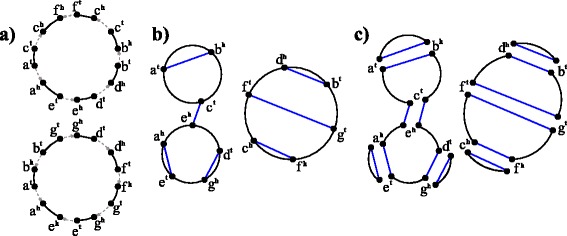
For an all-duplicated genome *A*=(−*a*−*b*+*g*+*d*+*f*+*g*+*e*)(−*a*+*c*−*f*−*c*−*b*−*d*−*e*) and an ordinary genome *R*=(−*a*−*b*−*d*−*g*+*f*−*c*−*e*), **a)** the genome graph $\mathfrak {G}(A)$; **b)** the contracted breakpoint graph $\hat {\mathfrak {G}}(A,R)$; **c)** a maximal *AR*-cycle decomposition of $\hat {\mathfrak {G}}(A,2R)$, which represents the ht-decomposition with respect to the clockwise orientation of *A*-cycles

For the sake of simplicity, we restrict our analysis to genomes with circular chromosomes. We represent a circular chromosome consisting of *n* genes as a graph cycle with *n* directed edges (encoding genes and their strands) alternating with *n* undirected edges (connecting the extremities of adjacent genes), called *P-edges* (Fig. 1 a). We label each directed edge with the corresponding gene *x*, and further label its tail and head endpoints with *x* ^*t*^ and *x* ^*h*^, respectively. For a genome *P* with *m* chromosomes, the *genome graph* $\mathfrak {G}(P)$ is formed by *m* such cycles representing the chromosomes of *P*. We remark that *P*-edges form a matching in $\mathfrak {G}(P)$, called *P-matching*.

A *Double-Cut-and-Join* (DCJ) (also called a *2-break*) operation breaks a genome at two positions and glue the resulting fragments in a new order, which model common types of genome rearrangements [23, 24]. A DCJ in genome *P* corresponds in $\mathfrak {G}(P)$ to the replacement of a pair of *P*-edges with a different pair of *P*-edges^2^ on the same set of four vertices.

**Fig. 2:**
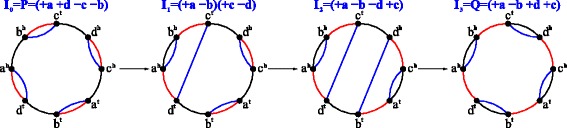
A shortest DCJ scenario transforming a genome *P*=(+*a*+*d*−*c*−*b*) (*red color*) into a genome *Q*=(+*a*−*b*+*d*+*c*) (*black color*). The intermediate genomes are shown in *blue color*

For genomes *P* and *Q* composed of the same set of genes, the *breakpoint graph* $\mathfrak {G}(P,Q)$ is defined as the superposition of genome graphs $\mathfrak {G}(P)$ and $\mathfrak {G}(Q)$ (Fig. 2 a). In other words, $\mathfrak {G}(P,Q)$ can be constructed by gluing the identically labeled directed edges in $\mathfrak {G}(P)$ and $\mathfrak {G}(Q)$. From now on, we will ignore directed edges and assume that the breakpoint graph $\mathfrak {G}(P,Q)$ consists only of (undirected) *P*-edges and *Q*-edges, forming *P*-matching and *Q*-matching. Then $\mathfrak {G}(P,Q)$ represents a collection of cycles consisting of edges alternating between *P*-edges and *Q*-edges, called *PQ-cycles* (or *QP-cycles*). Similarly, the breakpoint graph can be defined for three or more genomes [4].

A *DCJ scenario* between genomes *P* and *Q* is a sequence of DCJs transforming *P* into *Q*. A shortest such scenario has the following property:

#### **Lemma 1**

In a shortest DCJ scenario between genomes *P* and *Q*, each DCJ splits some *PQ*-cycle in their breakpoint graph into two and thus increases the number of *PQ*-cycles by one.

From Lemma 1, one can immediately get a formula for the *DCJ distance* (i.e., the length of a shortest DCJ scenario) between two genomes:

#### **Theorem 2**

The DCJ distance between genomes *P* and *Q* on *n* genes is given by the formula

$$d_{DCJ}(P,Q) = n - c(P,Q), $$

where *c*(*P,Q*) is the number of *PQ*-cycles in the breakpoint graph $\mathfrak {G}(P,Q)$.

### Whole genome duplications and contracted breakpoint graphs

The definition of breakpoint graph based on edge gluing can be easily extended to genomes with duplicated genes as follows. Let *A* be an all-duplicated genome and $\mathfrak {G}(A)$ be the corresponding genome graph. By the definition of an all-duplicated genome, the directed edges in the genome graph $\mathfrak {G}(A)$ come in pairs that are identically labeled (Fig. 1 a). By gluing edges in these pairs, we obtain the *contracted genome graph* $\hat {\mathfrak {G}}(A)$, where *A*-edges form cycles (since each vertex is incident to two *A*-edges), called *A-cycles*. For a doubled genome 2*R* resulted from a WGD^3^ of an ordinary genome *R*, the contracted genome graph $\hat {\mathfrak {G}}(2R)$ contains pairs of parallel *R*-edges, called 2*R*-edges. It is clear that 2*R*-edges form a matching in $\hat {\mathfrak {G}}(2R)$. Replacing 2*R*-edges with *R*-edges in $\hat {\mathfrak {G}}(2R)$ transforms it into the (contracted) breakpoint graph $\hat {\mathfrak {G}}(R) = \mathfrak {G}(R)$.

For an all-duplicated genome *A* and an ordinary genome *R* composed of the same genes, the *contracted breakpoint graph* $\hat {\mathfrak {G}}(A,R)$ (resp. $\hat {\mathfrak {G}}(A,2R)$) is defined as the superposition of $\hat {\mathfrak {G}}(A)$ and $\hat {\mathfrak {G}}(R)$ (resp. $\hat {\mathfrak {G}}(2R)$), and can be constructed in the same way as breakpoint graphs [9] (Fig. 1 b). The *A*-edges and *R*-edges in $\hat {\mathfrak {G}}(A,R)$ form *A*-cycles and *R*-matching, respectively.

The graph $\hat {\mathfrak {G}}(A,2R)$ can be decomposed into a collection of *AR*-cycles, called an *AR-cycle decomposition*. We remark that there exists an exponential number of *AR*-cycle decompositions of $\hat {\mathfrak {G}}(A,2R)$. Below, we describe two special types of *AR*-cycle decompositions. One is *maximal* *AR*-cycle decompositions, which have the maximum possible number of *AR*-cycles, denoted $c_{max}(\hat {\mathfrak {G}}(A,2R))$ (Fig. 1 c). Another type of *AR*-cycle decompositions is constructed as follows. For each *A*-cycle in $\hat {\mathfrak {G}}(A,2R)$, we fix some orientation. Then each *A*-edge becomes a directed edge. We decompose $\hat {\mathfrak {G}}(A,2R)$ into a collection of *AR*-cycles such that each *R*-edge in an *AR*-cycle connects the head of one *A*-edge and the tail of another. We call such *AR*-cycle decomposition an *ht-decomposition* of $\hat {\mathfrak {G}}(A,2R)$.

### GHP and RGGHP

Let us recall the formulation of the GHP and discuss the structure of its solutions.

#### **Problem**

For a given all-duplicated genome *A*, find an ordinary genome *R* minimizing *d*_*DCJ*_(*A*,2*R*).

In other words, the GHP asks for an ordinary genome *R* maximizing $c_{max}(\hat {\mathfrak {G}}(A,2R))$. Existence of such genome is guaranteed by the following theorem:

#### **Theorem 3**

For any all-duplicated genome *A*

$$\max\limits_{R}c_{max}(\hat{\mathfrak{G}}(A,2R))=n + k, $$

where maximum is taken over all ordinary genomes *R*, *n* is half the number of *A*-edges in $\hat {\mathfrak {G}}(A)$ (i.e., the number of distinct genes in *A*), and *k* is the number of even *A*-cycles in $\hat {\mathfrak {G}}(A)$.

It was shown in [9] that the maximum of $c_{max}(\hat {\mathfrak {G}}(A,2R))$ is achieved on genomes *R* such that $\hat {\mathfrak {G}}(A,R)$ is *R*-noncrossing as defined below.

For the graph $\hat {\mathfrak {G}}(A,R)$, an *R*-edge connecting vertices of distinct *A*-cycles is called *R-interedge*. An *R*-edge connecting vertices of same *A*-cycles is called *R-intraedge*. We represent vertices and edges of each *A*-cycle in $\hat {\mathfrak {G}}(A,R)$ as points and arcs on a circle, and draw all *R*-intraedges as straight chords inside these circles.

#### **Definition 4**

For a given all-doubled genome *A* and an ordinary genome *R*, the contracted breakpoint graph $\hat {\mathfrak {G}}(A,R)$ is *R-noncrossing* (Fig. 1 b) if its every connected component is formed by

- a single even *A*-cycle (i.e., *A*-cycle of even size) and noncrossing *R*-intraedges (as chords within the corresponding circle); or
- a pair of odd *A*-cycles (i.e., *A*-cycles of odd size) with single *R*-interedge and noncrossing *R*-intraedges.

While the condition of the graph $\hat {\mathfrak {G}}(A,R)$ being *R*-noncrossing guarantees that the genome *R* yields a solution to the GHP for an all-doubled genome *A*, this condition is not necessary, and there exist other genomes *R* solving the GHP (i.e., maximizing $c_{max}(\hat {\mathfrak {G}}(A,2R))$ as in Theorem 3). Namely, while in an *R*-noncrossing $\hat {\mathfrak {G}}(A,R)$ connected components with two odd *A*-cycles contain a single *R*-interedge, other solutions may have more than one *R*-interedge connecting such *A*-cycles. The following lemma establishes a correspondence between the GHP solutions and ht-decompositions of $\hat {\mathfrak {G}}(A,2R)$.

#### **Lemma 5**

Let an ordinary genome *R* be a solution to the GHP for an all-duplicated genome *A*. Then there exists an orientation of *A*-cycles such that the ht-decomposition of $\hat {\mathfrak {G}}(A,2R)$ is maximal.

The proof of Lemma 5 that requires the notions of non-orientable surfaces and gluings will be published elsewhere.

We remark that the maximal decomposition of an *R*-noncrossing graph $\hat {\mathfrak {G}}(A,R)$ proposed in [9] represents the ht-decomposition for the clockwise orientation of *A*-cycles (Fig. 1 c). More generally, Lemma 5 provides an important step towards a complete characterization and enumeration of the solutions to the GHP.

Since the solution space of the GHP is enormously large, one may restrict it by taking into account an additional genome and posing the following restricted problem:

#### **Problem**

Given an all-duplicated genome *A* and an ordinary genome *B*, find an ordinary genome *R* that is a solution to the GHP for *A* and minimizes *d*_*DCJ*_(*B,R*).

### Connection between IGMP and RGGHP

We recall the definition of an *intermediate genome* from [13] (Fig. 2):

#### **Definition 6**

An intermediate genome between two genomes is any genome appearing in a shortest DCJ scenario between them. In other words, a genome *I* is intermediate between genomes *P* and *Q* iff *d*_*DCJ*_(*P,I*)+*d*_*DCJ*_(*I,Q*)=*d*_*DCJ*_(*P,Q*).

Similarly to *R*-noncrossing contracted breakpoint graphs, for ordinary genomes *P*, *Q*, *I*, the breakpoint graph $\mathfrak {G}(P,Q,I)$ is called *I-noncrossing* if every its connected component is formed by a single *PQ*-cycle and noncrossing *I*-intraedges (as chords inside each *PQ*-cycle) (Fig. 2). The following theorem describes an important properties of intermediate genomes:

#### **Theorem 7**

For ordinary genomes *P* and *Q* on *n* genes, the following statements are equivalent:

1. a genome *I* is intermediate between genomes *P* and *Q*,
2. $\mathfrak {G}(P,Q,I)$ is *I*-noncrossing,
3. the total number of *PI*- and *QI*-cycles in $\mathfrak {G}(P,Q,I)$ equals *n*+*c*(*P,Q*).

Similarly to the GHP, one can restrict the solution space of the GMP to intermediate genomes and pose the following problem:

#### **Problem**

Given genomes *P*, *Q*, and an outgroup genome *R*, find an intermediate genome *I* between genomes *P* and *Q* that minimizes *d*_*DCJ*_(*R,I*).

From Theorem 7, one can observe that the intermediate genome *I* plays in the IGMP a similar role to those of the ordinary genome *R* in the GHP. Indeed, let *PQ* be an artificial all-duplicated genome formed by the union of genomes *P* and *Q*. Then the breakpoint graph $\mathfrak {G}(P,Q,I)$ can be viewed as the contracted breakpoint graph $\hat {\mathfrak {G}}(PQ,I)$, which has no odd *PQ*-cycles. If $\mathfrak {G}(P,Q,I)$ is *I*-noncrossing, then $\hat {\mathfrak {G}}(PQ,I)$ is also *I*-noncrossing, and $c_{max}(\mathfrak {G}(PQ,I)) = n + k$, where *k*=*c*(*P,Q*) is the number of cycles in $\hat {\mathfrak {G}}(PQ,I)$. More generally, the IGMP asks for a shortest DCJ scenario transforming the breakpoint graph $\mathfrak {G}(P,Q,R)$ into the breakpoint graph $\mathfrak {G}(P,Q,I)$ for some genome *I* such that $\mathfrak {G}(P,Q,I)$ is *I*-noncrossing. Thus, the IGMP can be viewed as a particular case of the RGGHP, where all cycles are even. We remark that Lemma 5 for the IGMP can be refined as follows: the ht-decomposition with respect to *any* orientation of *PQ*-cycles in $\mathfrak {G}(PQ,I)$ is maximal (since all *PQ*-cycles are even), and each cycle in this decomposition is either a *PI*-cycle or a *QI*-cycle.

Below we will show that both RGGHP and IGMP can be formulated within the framework of embedded graphs and polygon gluings.

## Methods

### Embedded graphs and glued surfaces

We recall the following definition from the topological graph theory:

#### **Definition 8**

A (2-cell) embedded connected graph *G*_*Σ*_ is a graph whose vertices and edges are points and arcs on a surface^4^ *Σ* such that

- the edges do not intersect (except at the vertices);
- the complement of *G*_*Σ*_ in *Σ* represents a collection of regions (called *faces*), and each face is a polygon.^5^

An embedded graph with *m* connected components is defined as the union $\{G^{(1)}_{\Sigma _{1}},G^{(2)}_{\Sigma _{2}},\dots, G^{(m)}_{\Sigma _{m}}\}$ of *m* connected embedded graphs $G^{(i)}_{\Sigma _{i}}$ (each on its own surface).

We remark that the complement of the connected embedded graph *G*_*Σ*_ in *Σ* can be viewed as the result of cutting *Σ* along the edges of *G*_*Σ*_. Conversely, *G*_*Σ*_ can be obtained by gluing the sides of its faces, which are polygons. Let us denote this collection of polygons by $\mathcal {P}$. Since each edge of *G*_*Σ*_ has two sides on *Σ*, the total number of sides in $\mathcal {P}$ is twice the number of edges in *G*_*Σ*_, and the edges of *G*_*Σ*_ define a (perfect) matching on the sides in $\mathcal {P}$. Since the surface *Σ* is orientable, we can orient sides of each face clockwise. Then the matched sides of $\mathcal {P}$ are glued in *G*_*Σ*_ head-to-tail.

**Fig. 3:**
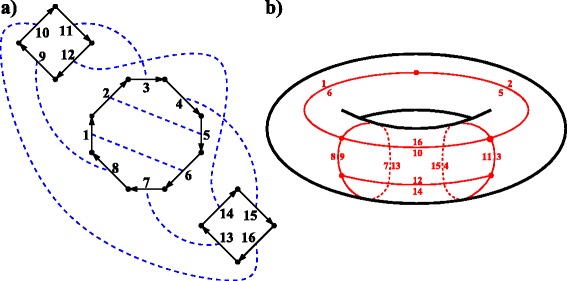
**a)** A collection $\mathcal {P}$ of three polygons (two 4-gons and one 8-gon) oriented clockwise, where blue dashed edges represent a matching on the sides in $\mathcal {P}$. **b)** The embedded graph *G*_*Σ*_ with *v*=5 vertices, *e*=8 edges, *f*=3 faces, and *g*(*Σ*)=1 (i.e., *Σ* is a torus) resulted from the oriented gluing of $\mathcal {P}$

For any collection of oriented polygons and a (perfect) matching on their sides (Fig. 3 a), we define the *orientable* gluing as the head-to-tail gluing of sides in each matched pair (Fig. 3 b). It is easy to see that the orientable gluing results in an embedded graph (possibly with several connected components). Unless stated otherwise, under polygon gluing we will understand the orientable gluing.

A polygon gluing according to a non-perfect matching is called *partial*. It results in an embedded graph *G*_*Σ*_ on a surface *Σ* *with boundary*. Connected components of the boundary are called *holes*. In this case, some edges of *G*_*Σ*_ represent glued pairs of sides, while the others represent non-glued sides and form holes.

For a connected embedded graph *G*_*Σ*_ with *v* vertices, *e* edges, and *f* faces, the Euler formula states that

1 $$ v-e+f+h(\Sigma) = 2-2g(\Sigma),  $$

where *h*(*Σ*) is the number of holes in *Σ* and *g*(*Σ*) is the topological genus (number of handles) of *Σ*. Unless *G*_*Σ*_ is the result of a partial gluing, we have *h*(*Σ*)=0.

### RGGHP and embedded graphs

We start with establishing a correspondence between contracted breakpoint graphs and embedded graphs.

Recall that for an all-duplicated genome *A*, the *A*-edges in $\hat {\mathfrak {G}}(A)$ form a collection of *A*-cycles. Let us fix some orientation *o* of these *A*-cycles. For each *A*-cycle with *k* edges, we assign a *k*-gon whose sides correspond to the cycle vertices (such that adjacent sides correspond to adjacent vertices). Then the sides of each polygon inherit labels from the corresponding cycle vertices, and the polygon itself inherits the orientation from the cycle. We denote the collection of these labeled oriented polygons by $\mathcal {P}_{o}(A)$.

**Fig. 4:**
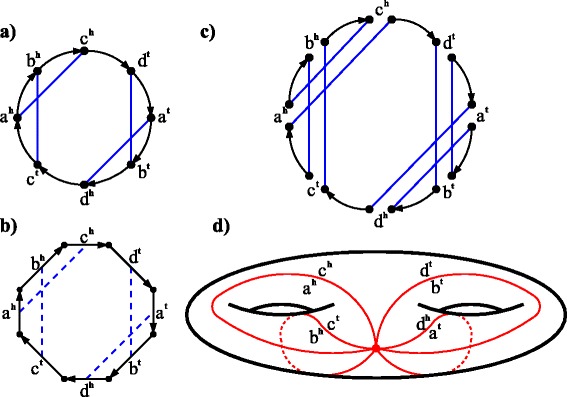
For an all-duplicated genome *A*=(+*a*+*c*−*b*−*d*)(+*a*−*b*)(+*c*+*d*) (*black edges*) and an ordinary genome *R*=(+*a*−*c*−*b*+*d*) (*blue edges*), **a)** the contracted breakpoint graph $\hat {\mathfrak {G}}(A,R)$, where the *A*-cycle is oriented clockwise; **b)** the polygon $\mathcal {P}_{o}(A)$ obtained from $\hat {\mathfrak {G}}(A,R)$, where the blue dashed lines represent a matching on the sides; **c)** the ht-decomposition of $\hat {\mathfrak {G}}(A,2R)$ consisting of a single *AR*-cycle; **d)** the gluing of $\mathcal {P}_{o}(A)$ resulting in an embedded graph *G*_*o*_(*A,R*) on a 2-torus (with *v*=1, *e*=4, *f*=1)

For an ordinary genome *R*, the *R*-edges in $\hat {\mathfrak {G}}(A,R)$ form an *R*-matching on the vertices of *A*-cycles and thus on the sides of $\mathcal {P}_{o}(A)$ (Fig. 4 a, b). It further defines a polygon gluing of $\mathcal {P}_{o}(A)$ resulting in an embedded graph *G*=*G*_*o*_(*A,R*) (Fig. 4 d).

#### **Lemma 9**

Let *A* be an all-duplicated genome, *R* be an ordinary genome, and *o* be some orientation of the *A*-cycles. Then the vertices of *G*_*o*_(*A,R*) are in one-to-one correspondence with the *AR*-cycles in the ht-decomposition of $\hat {\mathfrak {G}}(A,2R)$ with respect to the orientation *o*.

#### *Proof*

Recall that the vertices of $\mathcal {P}_{o}(A)$ correspond to the *A*-edges in $\hat {\mathfrak {G}}(A)$. Any vertex of *G* is an image of some vertices of $\mathcal {P}_{o}(A)$ under gluing. Let us prove that two vertices of $\mathcal {P}_{o}(A)$ are glued iff the corresponding *A*-edges belong to the same *AR*-cycle in the ht-decomposition of $\hat {\mathfrak {G}}(A,2R)$ (Fig. 4 c, d). Consider an arbitrary directed *A*-edge (*U*_1_,*U*_2_) in $\hat {\mathfrak {G}}(A)$. Let this edge belong to some subpath (*W*_1_,*V*_1_), {*V*_1_,*U*_1_}, (*U*_1_,*U*_2_), {*U*_2_,*V*_2_}, (*V*_2_,*W*_2_) in *AR*-cycle in the ht-decomposition of $\hat {\mathfrak {G}}(A,2R)$. Note that (*W*_1_,*V*_1_), (*U*_1_,*U*_2_), (*V*_2_,*W*_2_) are *A*-edges and {*V*_1_,*U*_1_}, {*U*_2_,*V*_2_} are (undirected) *R*-edges in $\hat {\mathfrak {G}}(A,2R)$. Then in *G*_*o*_(*A,R*) the side *V*_1_ is glued with *U*_1_ and the side *V*_2_ is glued with *U*_2_ (in head-to-tail fashion), and so the vertex corresponding to (*U*_1_,*U*_2_), which is the head of the side *U*_1_ and the tail of the side *U*_2_, is glued with the vertices corresponding to (*W*_1_,*V*_1_) (the tail of *V*_1_), and (*V*_2_,*W*_2_) (the head of *V*_2_). Conversely, since every gluing of matched sides implies gluing of vertices that correspond to *A*-edges from the same *AR*-cycle, vertices that correspond to *A*-edges from distinct *AR*-cycles can not be glued. By transitivity we obtain the statement of the lemma. □

#### **Lemma 10**

Let $\mathcal {P}$ be a set of *k* polygons with an even number of sides (even-gons) and 2*l* polygons with an odd number of sides (odd-gons). Then the graph obtained by gluing the sides of $\mathcal {P}$ contains at most *n*+*k* vertices, and this upper bound is achieved by the embedded graphs on *k*+*l* spheres.

#### *Proof*

Let $G = \{G^{(1)}_{\Sigma _{1}},G^{(2)}_{\Sigma _{2}},\dots, G^{(m)}_{\Sigma _{m}}\}$ be a result of some gluing of $\mathcal {P}$. By summing the Euler formula (1) over the connected components of *G*, we get that the total number of vertices in *G* is

$$v = n-(k+2l)+2m - 2\sum_{i=1}^{m} g(\Sigma_{i}), $$

where *n* is half the number of sides in $\mathcal {P}$ and *m* is a number of connected components in *G*. We remark that in order to maximize *v* we need to maximize *m* and minimize $\sum _{i=1}^{m} g(\Sigma _{i})$. The maximum value of *m* is *k*+*l*, and it is achieved iff each connected component of *G* is a result of gluing of either one even-gon or two odd-gons. The minimum value of *g*(*Σ*_*i*_) is achieved iff *Σ*_*i*_ is a sphere (so that *g*(*Σ*_*i*_)=0).

So, *G* has a maximal number of vertices (equal *n*+*k*) iff it has *k*+*l* connected components (each on a sphere). □

We remark that Lemmas 9 and 10 provide a topological interpretation of the GHP and essentially give a new proof of Theorem 3, which is much simpler than previous ones [25, 26].

#### **Lemma 11**

Let *A* be an all-duplicated genome, *R* be an ordinary genome, and *o* be some orientation of the *A*-cycles. Then a DCJ on the genome *R* corresponds in the embedded graph *G*_*o*_(*A,R*) to cutting two edges and gluing the resulting four sides in a new order (we call such operation a *DCJ-surgery*).

#### *Proof*

Let *R* ^′^ be the result of a DCJ on *R*. Then the *R*-matching and *R* ^′^-matching on the sides of $\mathcal {P}_{o}(A)$ differ only in two pairs of matched sides. The corresponding DCJ-surgery on *G*_*o*_(*A,R*) cuts the two pairs of sides matched in *R* and glues the resulted four sides according to *R* ^′^. □

Lemmas 9, 10, and 11 inspire us to pose the following problem:

#### **Problem**

Given an embedded graph *G*, find a shortest sequence of DCJ-surgeries that results in an embedded graph *G* ^′^ on a maximum number of spheres.

#### **Theorem 12**

1. The RGGHP for an all-duplicated genome *A* and an ordinary genome *B* is equivalent to the GSP for *G*_*o*_(*A,B*), where *o* is *some* orientation of *A*-cycles.
2. The IGMP for ordinary genomes *P*, *Q*, and an outgroup genome *T* is equivalent to the GSP for *G*_*o*_(*P* *Q,T*), where *o* is *any* orientation of *PQ*-cycles.

#### *Proof*

(1) Let *R* be a solution to the RGGHP for an all-duplicated genome *A* and an ordinary genome *B*. Let $\mathcal {S}$ be a shortest DCJ scenario $\mathcal {S}$ between *B* and *R*. By Lemma 5, there exists an orientation *o* of *A*-cycles such that the ht-decomposition of $\hat {\mathfrak {G}}(A,2R)$ is maximal. By Lemmas 9 and 10, *G*_*o*_(*A,R*) is an embedded graph on a maximum number of spheres. By Lemma 11, the DCJ scenario $\mathcal {S}$ corresponds to a shortest sequence of DCJ-surgeries transforming *G*_*o*_(*A,B*) into *G*_*o*_(*A,R*). Thus, the RGGHP for the genomes *A* and *B* is equivalent to the GSP for the embedded graph *G*_*o*_(*A,B*).

(2) Since all *PQ*-cycles in $\mathfrak {G}(PQ,R)$ are even, the ht-decomposition of $\mathfrak {G}(PQ,R)$ has a maximum number of *PR*- and *QR*-cycles for *any* orientation *o* of *PQ*-cycles. Thus, the IGMP for genomes *P*, *Q*, *T* is equivalent to the GSP for *G*_*o*_(*P* *Q,T*) with any orientation *o* of *PQ*-cycles. □

## Results

### Cardinality of the GHP solution space

Let us enumerate all the solutions to the GHP for a given all-duplicated genome *A*. For each solution *R*, there exists some orientation *o* such that *G*_*o*_(*A,R*) is an embedded graph on the maximum number of spheres. This inspires us to define a *maximal gluing* as a polygon gluing that results in an embedded graph on the maximum number of spheres. By Lemma 10, each connected component of this graph has either one even-gon face or two odd-gon faces.

We remark that there exists a method [27] that for any collection of polygons enumerate their gluings into an embedded graph on a surface of a given genus. Since the case of spheres is much easier than the general case, we can derive explicit formulas here.

#### **Lemma 13**

The number of ways to obtain a sphere by gluing the sides of a 2*k*-gon equals the *k*-th Catalan number $C_{k} = \frac {1}{k+1}\binom {2k}{k}$.

#### **Lemma 14**

The number of ways to obtain a single sphere by gluing the sides of a (2*n*+1)-gon and a (2*m*+1)-gon equals

$$T_{m,n}=\frac{2mn + m + n + 1}{m + n + 1}\binom{2m+1}{m} \binom{2n+1}{n}. $$

#### *Proof*

Let *G*_*Σ*_ be the result of some maximal gluing of a (2*n*+1)-gon and a (2*m*+1)-gon. By Euler formula (1), we have

$$v-e+2 = 2, $$

where *v* and *e* are the number of vertices and edges in *G*_*Σ*_, respectively. Since *v*=*e* and *G*_*Σ*_ is connected, there exists exactly one simple cycle in *G*_*Σ*_. Cutting *G*_*Σ*_ along edges of this cycle splits it into two connected components *G*_1_ and *G*_2_, each of which is an embedded graph on a sphere with one hole. So, the cycle is formed by all the edges whose sides belong to different faces. Since *G*_1_ and *G*_2_ contain non-glued sides, they represent the result of partial gluings of the (2*n*+1)-gon and the (2*m*+1)-gon, respectively. So, any maximal gluing can be obtained in the following way: for some *l*, *n*−*l* pairs of the (2*n*+1)-gon sides are glued and *m*−*l* pairs of the (2*m*+1)-gon sides are glued (transforming each of these polygons into a sphere with one hole), and the remaining 2*l*+1 sides from one polygon are glued with the remaining 2*l*+1 sides from the other (resulting in a sphere).

Let us enumerate all the maximal gluings of a (2*n*+1)-gon and a (2*m*+1)-gon. This is equivalent to enumeration of the pairs (*G*_1_,*G*_2_) and the ways to glue them into a sphere. Let 2*l*+1 be the length of the holes in *G*_1_ and *G*_2_. It is known [28] that there are $\binom {2k+1}{n-l}$ ways to obtain a sphere with one hole from a (2*k*+1)-gon by gluing *k*−*l* pairs of its sides. Hence, for each *l*, there exist $\binom {2m+1}{m-l}\binom {2n+1}{n-l}$ pairs (*G*_1_,*G*_2_). If *l*=0, then there is exactly one way to glue *G*_1_ and *G*_2_ together. If *l*>0, then there are 2(2*l*+1) ways to glue them into a single sphere (the factors 2*l*+1 and 2 account respectively for rotations and reflections of the holes in *G*_1_ and *G*_2_ with respect to each other). Combining these results together, we get that the number of maximal gluings of a (2*n*+1)-gon and a (2*m*+1)-gon equals

$$\begin{aligned} {}& \binom{2m+1}{m}\binom{2n+1}{n}\,+\,\sum_{l=1}^{n} 2(2l+\!1) \binom{2n+1}{n-l}\binom{2m+1}{m-l} \\ {}= & \binom{2m+1}{m} \binom{2n+1}{n} \left(1+\frac{2mn}{m+n+1}\right). \end{aligned} $$

□

Lemmas 13 and 14 lead to the following formula for the number of solutions to the GHP.

#### **Theorem 15**

For a given all-duplicated genome *A*, let 2*n*_1_,…,2*n*_*k*_ be the lengths of the even *A*-cycles and 2*m*_1_+1,…,2*m*_2*l*_+1 be the lengths of the odd *A*-cycles in $\hat {\mathfrak {G}}(A)$. Then the total number of ordinary genomes solving the GHP for *A* equals

$$\left(\prod_{i=1}^{k} C_{n_{i}}\right)\cdot \sum_{\mathcal{M}} \prod_{(i,j) \in \mathcal{M}} T_{m_{i},m_{j}}, $$

where the sum is taken over all matchings $\mathcal {M}$ on {1,2,…,2*l*}.

Since the IGMP represents a particular case of the RGGHP, where all cycles are even and the maximal gluings correspond to the intermediate genomes, Theorem 15 implies the following corollary (first observed in [13]):

#### **Corollary 16**

For given ordinary genomes *P* and *Q*, the number of intermediate genomes equals $\prod _{i=1}^{k} C_{n_{i}}$, where 2*n*_1_,…,2*n*_*k*_ are the lengths of the *PQ*-cycles in $\mathfrak {G}(P,Q)$.

### Solving the RGGHP in a particular case

**Fig. 5:**
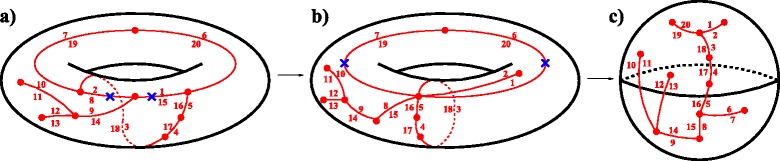
A shortest sequence of DCJ-surgeries (of length 2) transforming an embedded graph *G* on a torus (with *v*=9, *e*=10, *f*=1) into an embedded graph *H* on a sphere (with *v*=11, *e*=10, *f*=1). **a)** The embedded graph *G*; **b)** An (intermediate) embedded graph *G* ^′^ on a torus with *v*=9, *e*=10, *f*=1; **c)** The embedded graph *H*. Blue crosses mark edges on which the DCJ-surgeries operate

Theorem 12 shows that the RGGHP for given all-duplicated genome *A* and ordinary genome *B* is equivalent to the GSP for *G*=*G*_*o*_(*A,B*), where *o* is some orientation of *A*-cycles. In this section, we show how one can solve the GSP in the case of *G* being an embedded graph with a single face on a torus (Fig. 5 a).

#### **Lemma 17**

Let *G* be an embedded graph on a torus with one face. If *G* contains a simple cycle of length 2*l*, then *G* can be transformed into an embedded graph on a sphere with *l* DCJ-surgeries.

#### *Proof*

Consider a simple cycle of length 2*l* in *G*. If *l*>1, we apply a DCJ-surgery to two adjacent edges of this cycle such that the graph remains on a torus, thus decreasing the cycle length by 2 (Fig. 5 a, b). After *l*−1 such DCJ-surgeries, we obtain a graph on a torus with a cycle of length 2 (i.e., with *l*=1).

If *l*=1, we apply a DCJ-surgery that cuts the edges of this cycle, resulting in a sphere with two holes of length 2, and then glues each of these holes, resulting in a sphere. So, we have transformed *G* into an embedded graph on a sphere with *l* DCJ-surgeries. □

#### **Lemma 18**

Let *G* be an embedded graph on a torus with one face. If *G* contains two simple odd cycles that have the total length 2*l* and share exactly one vertex, then *G* can be transformed into an embedded graph on a sphere with *l* DCJ-surgeries.

#### *Proof*

Similarly to Lemma 17, we can apply *l*−1 DCJ-surgeries on *G* and obtain two loops (cycles of length 1) that share the vertex. We then apply a DCJ-surgery that cuts these loops, resulting in a sphere with a hole of length 4, and then glues this hole, resulting in a sphere. So, we have transformed *G* into an embedded graph on a sphere with *l* DCJ-surgeries. □

#### **Lemma 19**

Let *G* be an embedded graph on a surface with holes.

1. Let *g* be the genus of the surface of *G* and *G* ^′^ be obtained from *G* by gluing a pair of sides from different holes. Then the surface of *G* ^′^ has genus *g* ^′^=*g*+1.
2. If *G* has one face and can be glued into an embedded graph on a sphere, then *G* is an embedded graph on a sphere with holes of even length. Furthermore, all simple cycles in *G* are holes.

#### *Proof*

(1) Let *G* have *v* vertices, *e* edges, *f* faces and *h* holes. Let *C*_1_ and *C*_2_ be the holes that contain the pair of sides we are gluing. If at least one of the holes *C*_1_, *C*_2_ has length greater than 1, then *G* ^′^ has *v* ^′^=*v*−2 vertices, *e* ^′^=*e*−1 edges, *f* ^′^=*f* faces, and *h* ^′^=*h*−1 holes. If both *C*_1_ and *C*_2_ have length 1, then *G* ^′^ has *v* ^′^=*v*−1 vertices, *e* ^′^=*e*−1 edges, *f* ^′^=*f* faces, and *h* ^′^=*h*−2 holes. By the Euler formula (1), we have *g* ^′^=*g*+1 in both cases.

(2) Since *G* has one face, it results from a partial gluing of a polygon. Obviously, any partial gluing resulting in a sphere with holes of even length can be extended to a gluing resulting in a sphere. Let us prove that any other gluing can not be extended in such a way. Let *g* the genus of the surface of *G*. Consider a gluing of *G* into an embedded graph on a sphere. If *g*>0, such gluing does not exist, since the genus cannot be decreased by such gluing. Hence, *g*=0 and thus *G* is on a sphere with holes. If there are holes of odd lengths, then some side from one of these holes has to be glued with a side from some other hole, which would increase the genus. So, all holes must be of even length.

It remains to show that all the simple cycles in *G* are holes. Let *L* be the total length of the holes, and *v* and *e* be the number of vertices and edges of *G*, respectively. Consider the embedded graph *G* ^′^ resulting from contraction of the edges belonging to holes in *G*. Then *G* ^′^ is an embedded graph on a sphere, which has *v*+*h*−*L* vertices, *e*−*L* edges, and one face. From the Euler formula (1), we conclude that *G* ^′^ is a tree, thus all its edges are bridges. So, all edges of *G* except the edges belonging to the holes are bridges. □

#### **Theorem 20**

Let $\mathcal {S}$ be a shortest sequence of DCJ-surgeries transforming an embedded graph *G* with a single face on a torus into some embedded graph $\tilde {G}$ on a sphere. Then there exists a cycle of length $2|\mathcal {S}|$ in *G*.

#### *Proof*

Denote the face of *G* (and $\tilde {G}$) by *F*; clearly, *F* represents an even-gon. Let *M* and $\tilde {M}$ be the (perfect) matchings on the sides of *F* that define gluings resulting in *G* and $\tilde {G}$, respectively. Let *G* ^′^ be the result of a partial gluing of *F* defined by the (non-perfect) matching $M\cap \tilde {M}$. Then *G* ^′^ can be glued into each of *G* and $\tilde {G}$. Since $\tilde {G}$ is on a sphere, by Lemma 19 *G* ^′^ is an embedded graph on a sphere with holes of even length. Let 2*m* be the total length of these holes. Note that every non-glued edge in *G* ^′^ represents a side of an edge in *G* that should be cut by some DCJ-surgery from $\mathcal {S}$. Since each DCJ-surgery in $\mathcal {S}$ can create at most 4 non-glued sides, we have $4 |\mathcal {S}| \geq 2m$.

Let *b* be a bridge (i.e., an edge whose removal disconnects the graph) in *G* such that its sides *s*_1_, *s*_2_ are not glued in *G* ^′^. We will show that gluing of these sides into *b* in *G* ^′^ transforms this graph into another embedded graph $G^{\prime }_{b}$ still on a sphere with holes of even lengths. Since *b* is a bridge, *s*_1_ and *s*_2_ cannot belong to distinct holes in *G* ^′^. Let *C* be a hole in *G* ^′^ that contains both sides *s*_1_ and *s*_2_. In $G^{\prime }_{b}$, *C* is transformed into two holes *C*_1_ and *C*_2_ (possibly empty) connected by the edge *b*. It is clear that the lengths of *C*_1_ and *C*_2_ have the same parity. It remains to show that both lengths are even. Assume that they are odd. Since *b* is a bridge, no side of *C*_1_ is glued with a side of *C*_2_ in *G*. Hence, at least one side from *C*_1_ is glued with a side from a hole different from *C*_1_ and *C*_2_. Similarly, at least one side from *C*_2_ is glued with a side from a hole different from *C*_1_ and *C*_2_. By Lemma 19, gluing of two sides from different holes creates a handle, implying that *G* should contain at least two handles, a contradiction to *G* being an embedded graph on a torus (i.e., *G* has exactly one handle). Thus, both holes *C*_1_ and *C*_2_ in $G^{\prime }_{b}$ have even length, while the other holes in $G^{\prime }_{b}$ are inherited from *G* ^′^. This proves that $G^{\prime }_{b}$ is an embedded graph on a sphere with holes of even lengths.

Let *H* ^′^ be an embedded graph obtained from *G* ^′^ by gluing all non-glued sides of bridges in *G*. Then *H* ^′^ is on a sphere with holes of even lengths. Note that any edge in *G*, whose sides are non-glued in *H* ^′^, is not a bridge and thus belongs to some simple cycle in *G*.

Consider a gluing of *H* ^′^ into *G*. A handle in *G* can be created by gluing either two sides from distinct holes, say *C*_1_ and *C*_2_, or from one hole, say *C*, in *H* ^′^. In the former case, sides from *C*_1_ and *C*_2_ cannot be glued with sides from any other holes (otherwise, there would be at least two handles in *G* by Lemma 19). The sides from *C*_*i*_ (*i*=1,2) cannot be glued with any other side from *C*_*i*_, since this would result in a bridge missing in *H* ^′^. Thus, the sides from *C*_1_ and *C*_2_ are glued into edges that form a simple cycle in *G* of length 2*l* (equal the length of each *C*_*i*_). Since |*C*_1_|+|*C*_2_|≤2*m*, we have 4*l*≤2*m*. In the latter case, we claim that the edges resulted from gluing of the sides of *C* form two simple cycles in *G*, which share a vertex. Indeed, let 2*p* be the length of *C*, and *H* ^′^ have *V*+2*p* vertices, *E*+2*p* edges, and *h* holes. After gluing the sides of *C* (as in *G*), we obtain a graph on a torus with *V*+*v* vertices, *E*+*p* edges, and *h*−1 holes, where *v* vertices and *p* edges are obtained from vertices and edges in *C* and form a (possibly non-simple) cycle $\tilde {C}$ in *G*. By the Euler formula (1), we have *v*=*p*−1, and so $\tilde {C}$ is formed by two simple cycles sharing a vertex. Clearly, either one of these simple cycles has an even length, or $\tilde {C}$ itself has an even length. Let the even cycle have the length 2*l*, then 4*l*≤2*p*≤2*m*.

Since $\mathcal {S}$ transforms *G* into $\tilde {G}$, the above analysis implies that some cycle of length 2*l* should be cut by DCJ-surgeries from $\mathcal {S}$. Hence, $4l \leq 2m \leq 4|\mathcal {S}|$. By Lemmas 17 and 18, we have $|\mathcal {S}| \leq l$. Thus, $|\mathcal {S}| = l$, and there exists a cycle of length $2|\mathcal {S}|=2l$ in *G*. □

Theorem 20 inspires us to design the following algorithm for solving the RGGHP for given all-duplicated genome *A* and ordinary genome *B* such that the contracted breakpoint graph $\hat {\mathfrak {G}}(A,B)$ corresponds to an embedded graph on a torus with a single face (hence, $\hat {\mathfrak {G}}(A,B)$ has a single *A*-cycle of even length).

1. Construct $\hat {\mathfrak {G}}(A,B)$ and fix an arbitrary^6^ orientation *o* on its *A*-cycle.
2. From $\hat {\mathfrak {G}}(A,B)$ and *o*, construct the embedded graph *G*_*o*_(*A,B*).
3. Using the breadth-first search (BFS) starting at each vertex in *G*_*o*_(*A,B*), find a shortest even cycle *C* in *G*_*o*_(*A,B*).
4. Construct a sequence of |*C*|/2 DCJ-surgeries that cut the edges of *C* and transform *G*_*o*_(*A,B*) into an embedded graph on a sphere.
5. Apply the corresponding DCJs to the genome *B* and return the resulting genome as a solution to the RGGHP.

We remark that our algorithm runs in polynomial time. Indeed, the most time-consuming step is the BFS starting at each vertex of *G*_*o*_(*A,B*). Since in *G*_*o*_(*A,B*) the number of edges equals *n*=|*B*|=|*A*|/2 and the number of vertices equals *n*−1, this step runs in *O*(*n* ^2^) time.

## Discussion

In the present study we establish a somewhat unexpected link between the restricted variants of genome median and halving problems and embedded graphs. We provide a new simple proof for existence of the GHP solutions as well as completely describe the structure of the GHP solution space and determine its cardinality. We also show how the topological framework can be applied for solving the restricted guided genome halving problem (and the intermediate genome median problem) in a particular case. In further development we plan to address the topological problem of an embedded graph surgery (GSP) on an arbitrary orientable surface (i.e., a sphere with handles), which may provide better heuristic solutions for the RGGHP and IGMP.

We remark that similar topological interpretations exist for other comparative genomics problems and can provide intuition for their solution. For example, analysis of non-orientable surfaces (such as Klein bottle) seems to be relevant to the *double distance problem* asking for a maximal cycle decomposition of the contracted breakpoint graph of a given all-duplicated genome and an ordinary genome. Also, embedded graphs on surfaces with boundaries (holes) can be related to models including genome rearrangements along with gene insertions and deletions [29, 30].

## Endnotes

^1^ Some studies base their analysis on synteny blocks rather than genes. We will use the term “gene” to refer to an actual gene or a synteny block.

^2^ Here we view genome *P* as being transformed and *P*-edges as changing.

^3^ A WGD event can simultaneously duplicate each circular chromosome in genome *Q* either into a single circular chromosome or into two identical circular chromosomes, which have the same contracted genome graph [25]. We assume that a doubled genome 2*R* may contain duplicated chromosomes of both types.

^4^ Under a surface we understand a 2-dimensional compact orientable manifold without boundary (e.g., a sphere or a torus). We distinguish surfaces up to homeomorphisms.

^5^ Under a polygon (*n*-gon) we understand a topological disc, whose boundary is formed by a collection of *n* sides.

^6^ There exist two orientations of the *A*-cycle in $\hat {\mathfrak {G}}(A,B)$, both corresponding to the same ht-decomposition.
